# Temporal Association Between ChatGPT-Generated Diarrhea Synonyms in Internet Search Queries and Emergency Department Visits for Diarrhea-Related Symptoms in South Korea: Exploratory Study

**DOI:** 10.2196/65101

**Published:** 2025-05-22

**Authors:** Jinsoo Kim, Ansun Jeong, Juseong Jin, Sangjun Lee, Do Kyoon Yoon, Soyeoun Kim

**Affiliations:** 1 Department of Emergency Medicine Hanyang University College of Medicine Seoul Republic of Korea; 2 Department of Preventive Medicine Hanyang University College of Medicine Seoul Republic of Korea; 3 Department of Urology Seoul National University Hospital Seoul Republic of Korea; 4 Department of Preventive Medicine Seoul National University College of Medicine Seoul Republic of Korea; 5 Integrated Major in Innovative Medical Science Seoul National University Graduate School Seoul Republic of Korea; 6 Cancer Research Institute Seoul National University Seoul Republic of Korea; 7 Department of Data Science Research Innovative Medical Technology Research Institute Seoul National University Hospital Seoul Republic of Korea; 8 Biomedical Research Institute Seoul National University Hospital Seoul Republic of Korea

**Keywords:** emergency room, synonyms, diarrhea, symptoms, relative search volumes

## Abstract

**Background:**

Diarrhea, a common symptom of gastrointestinal infections, can lead to severe complications and is a major cause of emergency department (ED) visits.

**Objective:**

This study explored the temporal association between internet search queries for diarrhea and its synonyms and ED visits for diarrhea-related symptoms.

**Methods:**

We used data from the National Emergency Department Information System (NEDIS) and NAVER (Naver Corporation), South Korea’s leading search engine, from January 2017 to December 2021. After identifying diarrhea synonyms using ChatGPT, we compared weekly trends in relative search volumes (RSVs) for diarrhea, including its synonyms and weekly ED visits. Pearson correlation analysis and Granger causality tests were used to evaluate the relationship between RSVs and ED visits. We developed an Autoregressive Integrated Moving Average with Exogenous Variables (ARIMAX) model to further predict these associations. This study also examined the age-based distribution of search behaviors and ED visits.

**Results:**

A significant correlation was observed between the weekly RSV for diarrhea and its synonyms and weekly ED visits for diarrhea-related symptoms (ranging from 0.14 to 0.51, *P*<.05). Weekly RSVs for diarrhea synonyms, such as “upset stomach,” “watery diarrhea,” and “acute enteritis,” showed stronger correlations with weekly ED visits than weekly RSVs for the general term “diarrhea” (ranging from 0.20 to 0.41, *P*<.05). This may be because these synonyms better reflect layperson terminology. Notably, weekly RSV for “upset stomach” was significantly correlated with weekly ED visits for diarrhea and acute diarrhea at 1 and 2 weeks before the visit (*P*<.05). An ARIMAX model was developed to predict weekly ED visits based on weekly RSVs for diarrhea synonyms with lagged effects to capture their temporal influence. The age group of <50 years showed the highest activity in both web-based searches and ED visits for diarrhea-related symptoms.

**Conclusions:**

This study demonstrates that weekly RSVs for diarrhea synonyms are associated with weekly ED visits for diarrhea-related symptoms. By encompassing a nationwide scope, this study broadens the existing methodology for syndromic surveillance using ED data and provides valuable insights for clinicians.

## Introduction

The primary causes of admission to emergency departments (EDs) in South Korea [1] and the United States [2] are “gastroenteritis and colitis of infectious and unspecified origin.” Diarrhea, a common symptom of gastrointestinal infections, can lead to severe dehydration and hypovolemic shock [3]. The Global Burden of Disease Study reports approximately 2.39 billion annual cases of diarrhea worldwide, resulting in approximately 480,000 deaths among children younger than 5 years in 2019 [4].

The internet is a critical tool for analyzing health information–seeking behaviors, including those related to diarrhea [5]. Web-based surveillance tools effectively analyze human behavior [6], gauge disease prevalence [7-11], and forecast infectious disease outbreaks [12,13]. Previous applications of artificial intelligence (AI) models in public health surveillance used models that analyzed data from various sources, including electronic health records, social media, and travel data, to predict future incidences of respiratory infections, such as COVID-19 and influenza, but not gastrointestinal infections [14]. Gastrointestinal symptoms, such as abdominal pain, diarrhea, and vomiting, are indicators for identifying symptom outbreaks and monitoring public health trends [15]. However, many symptoms related to diarrhea have been overlooked, and identifying appropriate keywords for gastrointestinal symptoms remains challenging [9,16].

Many studies have explored large language models and their applications in gastrointestinal diseases. However, only a limited number of studies have focused on monitoring gastrointestinal symptoms through web-based surveillance [17,18]. Advancements in AI have shown that large language models such as OpenAI’s ChatGPT can understand human language and address health-related research problems. Compared to traditional expert-driven or systematic review–based methods [17-19], ChatGPT is particularly well-suited for generating synonyms, as it is trained on large-scale natural language data and can recognize colloquial, idiomatic, and context-specific expressions commonly used by the general public [20-22]. This capacity enables the generation of search terms that more accurately reflect how individuals describe symptoms in real-world web-based searches. This study aimed to investigate the association between real-time data on diarrhea-related symptoms obtained from the National Emergency Department Information System (NEDIS) and relative search volumes (RSVs) of diarrhea and its synonyms on NAVER, South Korea’s most widely used search engine. By examining these relationships, the study seeks to enhance the potential of web-based surveillance for predicting gastrointestinal outbreaks and informing ED resource allocation.

## Methods

### Data Source and Setting

The NEDIS was established in 2003 under the Emergency Medical Service Act to assess the quality of care delivered in EDs across South Korea [23]. Data from each patient visit is automatically transmitted from the visited EDs to a central government server within 2 to 14 days after the patient leaves the ED or hospital [24]. The transmitted data includes patient demographics (sex, age, and insurance type), primary complaints, vital signs, triage details, ED visit outcomes, and diagnosis codes according to the *Korean Standard Classification of Diseases and Causes of Death 7th edition* [25,26]. Between January 2017 and December 2021, the NEDIS database included information on 420,819 ED visits for diarrhea-related symptoms. NAVER was selected as the primary search engine for this study not only because it is the most dominant platform in South Korea, but also due to its overwhelming market share relative to alternatives. NAVER accounted for approximately 70% of the domestic search engine market, far surpassing Google, Daum, and other platforms [9,27]. This high penetration ensures that NAVER-based data should reflect the majority of population-level search behaviors in Korea, thereby providing a robust foundation for syndromic surveillance. NAVER Data Lab offers a search term trend service that tracks the longitudinal trend of RSVs for various topics, starting from January 2016. RSVs were standardized, with the highest search volume for a subject term set to 100 during a specified period [28,29], resulting in RSVs represented as relative percentages. This study used weekly trend data from NAVER Data Lab, identifying 4 Korean search terms for diarrhea and their synonyms based on GPT-4 (Figure S1 and Table S1 in Multimedia Appendix 1). This study was granted a waiver by the Institutional Review Board of Seoul National University Hospital (IRB No E-2406-089-1544).

### Diarrhea-Related Symptoms From NEDIS Data

In the NEDIS, Unified Medical Language System (UMLS) codes were collected for chief complaints, with up to 3 complaints recorded per visit. Based on the chief complaint, we selected UMLS-coded symptoms indicative of diarrhea (C0011991), acute diarrhea (C0740441), watery diarrhea (C0239182), and vomiting with diarrhea (C0474496). Based on the data from the NEDIS database, 420,819 visits were for diarrhea-related symptoms between January 2017 and December 2021. The data were divided into 5 age groups to compare searching and ED visits: 0-18 years (83,425 visits), 19-29 years (67,638 visits), 30-39 years (56,513 visits), 40-49 years (44,223 visits), and >50 years (169,020 visits).

### Data Acquisition of Diarrhea Synonyms From NAVER

GPT-4 was used on March 5, 2024, to identify Korean synonyms for diarrhea (Figure S1 in Multimedia Appendix 1). To identify synonyms related to diarrhea for use in this study, we used ChatGPT to generate an initial list of potential terms. This list was then reviewed collaboratively by all authors, including physicians, to ensure that the terms met the following criteria: (1) relevance to NEDIS data: the terms needed to correspond to chief complaints recorded in the NEDIS database; (2) RSVs viability: each term’s weekly RSV had to consistently exceed zero. Through this rigorous process, we selected 4 terms that aligned with these criteria, ensuring they were both clinically relevant and representative of real-world users’ search behavior. A total of 3 synonyms were chosen for comparison with the ED visits for diarrhea-related symptoms based on UMLS codes—upset stomach, watery diarrhea, and acute enteritis. An upset stomach involves digestive discomfort, including nausea, bloating, pain, and diarrhea. Watery diarrhea refers to loose liquid bowel movements with high water content. Acute enteritis means sudden inflammation of the small intestine, typically causing diarrhea and abdominal pain. These terms were used to collect weekly RSVs via the NAVER API from January 2017 through December 2021 (Table S1 in Multimedia Appendix 1). Following the guideline that acute diarrhea lasts for less than 14 days [30], we subdivided the lag times into 1- and 2-week intervals to account for the temporal relationship between RSVs and ED visits for acute diarrhea. To validate the synonyms generated by ChatGPT, we compared the RSVs of the general term “diarrhea” with those of the ChatGPT-suggested synonyms (“upset stomach,” “watery diarrhea,” and “acute enteritis”) during the study period. We hypothesized that valid synonyms would exhibit similar search patterns, evidenced by strong correlations. The analysis statistically revealed significant correlations (ranging from 0.4 to 0.7; *P*<.05) using the Pearson method, supporting the validity of these synonyms in representing similar search behaviors.

### Statistical Analyses

To compare the trends between the mean of the total weekly RSVs, including diarrhea and its synonyms, and total weekly ED visits for diarrhea-related symptoms, we calculated total weekly diarrhea-related symptoms based on chief complaints. To calculate the mean of the total weekly RSV, we averaged weekly RSVs for diarrhea and its synonyms obtained from the NAVER API. The correlation between weekly ED visits for diarrhea-related symptoms and weekly RSVs for diarrhea and its synonyms was evaluated using Pearson correlation analysis. The Granger causality test was used to determine the temporal relationship between weekly RSVs, 1 and 2 weeks before weekly ED visits for diarrhea-related symptoms. An Autoregressive Integrated Moving Average with Exogenous Variables (ARIMAX) model was developed to predict the associations between weekly RSV for diarrhea synonyms and weekly ED visits for diarrhea-related symptoms. The ARIMAX model extends the ARIMA framework by incorporating exogenous variables to improve prediction accuracy [31]. The model is specified as ARIMAX(*p*, *d*, *q*)(*P*, *D*, *Q*)[*s*], where:

- *p* and *P* denote the nonseasonal and seasonal autoregressive orders, respectively,
- *d* and *D* represent the nonseasonal and seasonal differencing orders, respectively,
- *q* and *Q* indicate the nonseasonal and seasonal moving average orders, respectively, and
- *s* corresponds to the seasonal period.

Unlike ARIMA, ARIMAX allows for the incorporation of external variables (exposures) to predict the outcome variable. In this study, weekly RSV for diarrhea synonyms, including upset stomach, watery diarrhea, and acute enteritis, were used as exposures to predict weekly ED visits for diarrhea and watery diarrhea as outcomes. The ARIMAX model was fitted automatically by selecting the optimal *p*, *d*, *q, P, D, Q*, and s parameters using the Hyndman-Khandakar algorithm [32]. This algorithm first determines the appropriate degree of differencing (*d*) by applying repeated KPSS (Kwiatkowski-Phillips- Schmidt-Shin) tests within the range 0-2 [33]. It then uses a stepwise search to find the AR and MA orders (*p* and *q*), as well as any seasonal components (*P, Q*, and seasonal period *s*), that minimize the Corrected Akaike Information Criterion (AICc). The procedure begins by fitting several initial candidate models, selects the best-performing model (lowest AICc) as the “current model,” and iteratively refines it by adjusting model orders and the inclusion or exclusion of a constant term. This process continues until no further improvement in AICc is achieved, thus identifying the final model specification. The ARIMAX model was first fitted on each weekly RSV for diarrhea synonyms up to 2020, to establish the association between weekly RSVs for diarrhea synonyms and weekly ED visits for diarrhea and watery diarrhea. Subsequently, the model was used to forecast weekly ED visits in 2021, using RSVs for diarrhea synonyms. The predicted values were then compared with the observed ED visits for diarrhea and watery diarrhea to evaluate the model's predictive performance. Accuracy metrics, including mean absolute percentage error and symmetric mean absolute percentage error, were calculated to quantify prediction error. To explore the potential lagged effects of weekly RSV for diarrhea synonyms on ED visits for diarrhea and watery diarrhea, sensitivity analyses were conducted by introducing lag times of 0, 1, and 2 weeks. In addition, we compared the age group-based distribution between ED visits for diarrhea-related symptoms and RSVs for diarrhea and its synonyms. All reported *P* values were 2-sided with a type I error threshold of *α*<.05, and were considered statistically significant. Statistical analyses were performed using SAS version 9.4 (SAS Institute) and “forecast” R packages (R software, version 4.4.1 R Core Team) [32].

## Results

**Figure 1:**
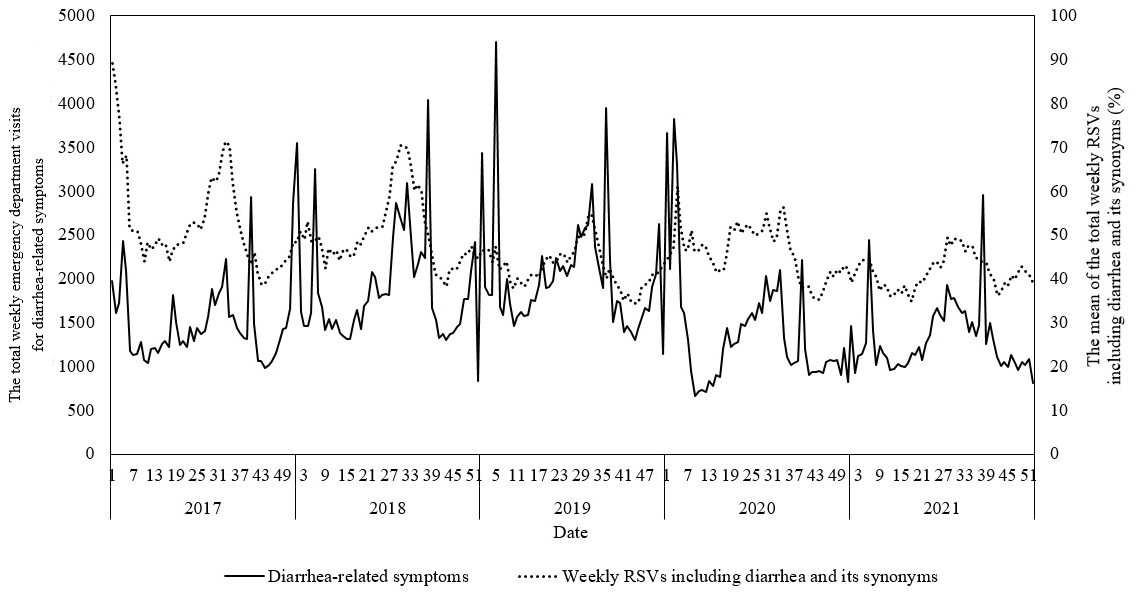
Trends in the total weekly emergency department visits for diarrhea-related symptoms from NEDIS and the mean weekly relative search volumes, including diarrhea and its synonyms from NAVER, from January 2017 to December 2021. ED: emergency department; NEDIS: National Emergency Department Information System; RSV: relative search volume.

In Figure 1 the total weekly ED visits for diarrhea-related symptoms from the NEDIS and the mean of the total weekly RSVs, including diarrhea and its synonyms from NAVER, from January 2017 to December 2021 has been illustrated. The trend in the mean of the total weekly RSVs, including diarrhea and its synonyms, closely mirrored the trend in total weekly ED visits for diarrhea-related symptoms.

Table S2 in Multimedia Appendix 1 shows the correlation between weekly ED visits for diarrhea-related symptoms and weekly RSVs for diarrhea and its synonyms. Weekly ED visits for diarrhea had a significantly higher correlation with weekly RSVs for “upset stomach,” “watery diarrhea,” and “acute enteritis” than with those for “diarrhea” (*r*=0.41, *P*<.001; *r*=0.30, *P*<.001; *r*=0.22, *P*<.001, respectively, versus *r*=0.20, *P*<.001). The weekly RSV for “upset stomach” was significantly correlated with weekly ED visits for diarrhea at lags of 1 and 2 weeks (*P*<.05). For weekly RSV for “acute enteritis,” only a 2-week previous correlation was significant (*P=.*02). Weekly RSV for “watery diarrhea” strongly correlated with weekly ED visits for watery diarrhea (r=0.51, *P<.*001), with significant correlations at 1 and 2 weeks before the visits (1 week previous, *P*=.002; 2 weeks previous *P=.*009). Weekly ED visits for vomiting with diarrhea did not show a significant correlation with weekly RSVs, except for “upset stomach” (*r*=0.21, *P*<.001; 1 week previous, *P*=.04).

**Figure 2:**
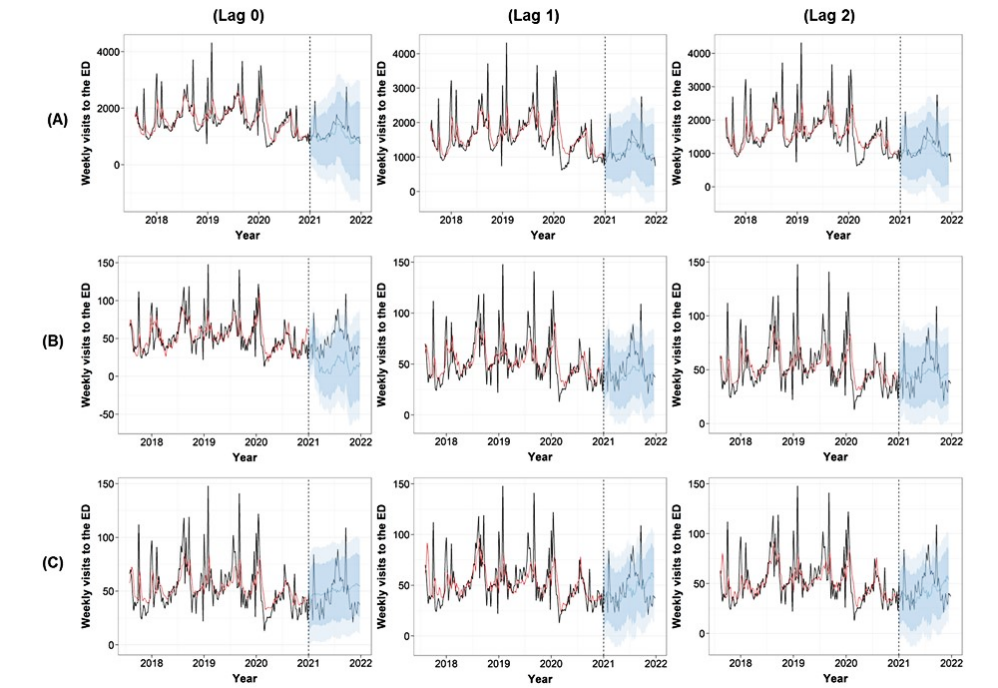
Actual values (black line), fitted values (red line), and predicted values (blue line with 95% CIs) from Autoregressive Integrated Moving Average with Exogenous models showing the associations between weekly relative search volumes for diarrhea synonyms—upset stomach, watery diarrhea, and acute enteritis—and weekly emergency department visits for diarrhea and watery diarrhea across lag times of 0 to 2 weeks. (A) Weekly relative search volumes for “upset stomach” associated with weekly emergency department visits with diarrhea. (B) Weekly relative search volumes for “watery diarrhea” associated with weekly emergency department visits with watery diarrhea. (C) Weekly relative search volumes for “acute enteritis” associated with weekly emergency department visits with watery diarrhea. ARIMAX: Autoregressive Integrated Moving Average with Exogenous variables; ED: emergency department; RSVs: relative search volumes.

Weekly RSVs for “upset stomach,” “watery diarrhea,” and “acute enteritis” appear significantly associated with weekly ED visits for diarrhea and watery diarrhea across lag times of 0 to 2 weeks in the ARIMAX models (Table S3 in Multimedia Appendix 1 and Figure 2). The ARIMAX models with the best fit and smallest error metrics based on root-mean-square error and symmetric mean absolute percentage error vary by weekly RSVs and lag times. In weekly RSV for “upset stomach” and weekly ED visits due to diarrhea, the best-fitting model is ARIMAX(0,1,3; 0,0,0) [52] at Lag 2, with a coefficient of 16.215 (SE 4.813, *P<*.001; Figure 2A). In weekly RSV for “watery diarrhea” and weekly ED visits due to watery diarrhea, ARIMAX(1,0,1; 0,0,0) [52] at Lag 1 demonstrates a strong association with a coefficient of 1.848 (SE 0.153, *P*<.001; Figure 2B). Similarly, association between weekly RSV for “acute enteritis” and weekly ED visits due to watery diarrhea shows significant lag times at Lag 1 and Lag 2 using ARIMAX(3,0,2; 0,0,0) [52], with coefficients of 1.123 (SE 0.141; *P*<.001) and 1.034 (SE 0.200; *P*<.001; Figure 2C).

**Figure 3:**
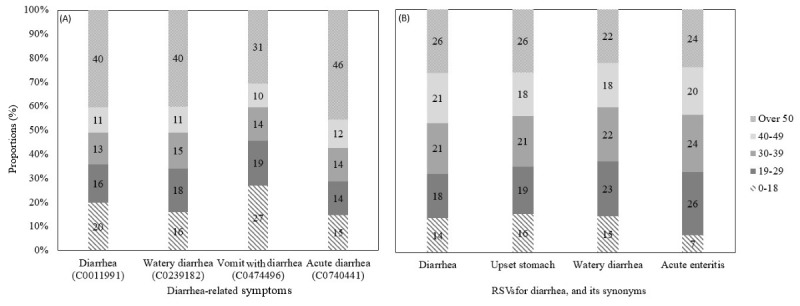
Age group–based distribution of relative search volumes for diarrhea and its synonyms from NAVER and emergency department visits for diarrhea-related symptoms from the National Emergency Department Information System, from January 2017 to December 2021. (A) National Emergency Department Information System (B) NAVER. ED: emergency department; NEDIS: National Emergency Department Information System; RSV: relative search volume.

In Figure 3 the age group-based distribution of RSVs for diarrhea and its synonyms from NAVER and ED visits for diarrhea-related symptoms from NEDIS were presented, from January 2017 to December 2021. The age group with the highest proportion of RSV for diarrhea and its synonyms and ED visits for diarrhea-related symptoms was over 50 years old. The RSVs for “diarrhea” and “upset stomach” each accounted for 26% of total search volume, which was higher than that of other diarrhea synonyms. In comparison, the proportions of ED visits for acute diarrhea and watery diarrhea were 46% and 40%, respectively.

## Discussion

### Principal Findings

Our study, conducted from January 2017 to December 2021, revealed significant correlations between the mean of the total weekly RSVs, including diarrhea and its synonyms, and total weekly ED visits for diarrhea-related symptoms. The trend in mean total weekly RSVs closely mirrored that of the total weekly ED visits. Notably, the correlations between weekly ED visits and weekly RSVs were stronger for synonyms such as “upset stomach,” “watery diarrhea,” and “acute enteritis” than for the general term “diarrhea.” The weekly RSV for “upset stomach” was significantly correlated with weekly ED visits for diarrhea at lags of 1 and 2 weeks. Similarly, the weekly RSVs for “watery diarrhea” and “acute enteritis” were significantly correlated with ED visits for watery diarrhea at lags of 1 and 2 weeks. However, weekly ED visits with vomiting and diarrhea showed no significant relationship with weekly RSVs, except those with “upset stomach.” ARIMAX models further confirmed the lag times between weekly RSVs for diarrhea synonyms and weekly ED visits, demonstrating strong predictive performance across weekly RSVs and lag times. Along with that, search engine users older than 50 years showed the highest proportion of both RSVs and ED visits for diarrhea-related symptoms. Among the search terms, “diarrhea” and “upset stomach” each accounted for 26% of the total search volume. In comparison, ED visits for acute and watery diarrhea accounted for 46% and 40%, respectively.

Many people prefer to search the internet for health information before visiting EDs [34]. A previous study has shown that the relative frequency of searches for gastrointestinal symptoms, including diarrhea, closely mirrored the changing incidences of cases in large inpatient datasets [17]. Our findings align with those of previous studies, indicating that search trends for diarrhea symptoms reflect ED visit trends. Google Trends automatically compiles search terms related to each symptom and other related search terms; however, the exact compilation process and methodology are not publicly disclosed. Studies in Korea have shown that representative keywords for foodborne diseases, such as “Seol-sa” (diarrhea) [9], can track population-level changes in the incidence of diarrhea through search volumes [18]. This finding supports the idea that search engine data can be used to monitor ED visits.

Our results showed that weekly ED visits for diarrhea had a higher correlation with weekly RSVs for diarrhea synonyms, including “upset stomach”, “watery diarrhea”, and “acute enteritis”, than with those for “diarrhea”. Adults suffering from diarrhea, particularly acute forms, commonly seek medical evaluation in EDs [35]. The criteria for acute diarrhea include volume depletion and 6 or more stools in 24 hours, making “watery diarrhea” a more relevant term for acute diarrhea than chronic diarrhea [36,37]. This explains why weekly RSVs for “watery diarrhea” are related to weekly ED visits for diarrhea-related symptoms, as acute diarrhea requires immediate medical evaluation [38]. Similarly, “upset stomach” and “acute enteritis” can be the terms used for acute diarrhea, supporting the observation that searches for these keywords precede ED visits. In addition, acute diarrhea lasts for less than 14 days, while diarrhea lasting more than 14 days is termed “persistent”, and those lasting over 1 month are termed “chronic” [30]. While vomiting episodes are typically short in duration, they do not correlate well with internet searches conducted 1–2 weeks before ED visits. Therefore, our results showed that weekly RSVs indicating acute diarrhea became notable within 1–2 weeks, whereas weekly ED visits for vomiting with diarrhea did not show a significant relationship with weekly RSVs for diarrhea and its synonyms. In addition, the weak or nonsignificant correlations between weekly ED visits for vomiting with diarrhea, and weekly RSV for diarrhea and its synonyms may reflect a discrepancy between actual symptom presentations in ED visits and real-world search behaviors. While patients who visit the ED with “vomiting with diarrhea” are experiencing both symptoms concurrently, most internet users tend to search for only one dominant symptom at a time rather than enter queries that combine multiple symptoms [39]. As a result, compound search phrases that include both vomiting and diarrhea are rarely used, which may lead to lower relative search volumes and weaker correlations with ED visit data, despite their clinical relevance.

A previous study conducted in Germany reported that individuals aged 36-55 years were the most active users of search engines for health-related information, while those older than 56 years demonstrated the lowest levels of engagement [40]. In contrast, South Korea exhibits one of the highest internet penetration rates globally, including among older adults, with more than 90% of individuals aged 50 years and older using the internet as of 2021. Recent national statistics show that 70.2% of Korean internet users search for health and medical information, with comparable rates among men (70.7%) and women (69.7%). Age-stratified data further indicate that individuals in their 30s had the highest rate of health information-seeking (80.3%), followed closely by those in their 40s (79.9%), 50s (78%), and 60s (75.7%) [41]. This upward trend in digital engagement among older adults is supported by previous research showing a narrowing digital health divide and increasing internet use for health-related purposes in older populations [42,43]. In the context of our study, individuals older than 50 years not only exhibited the highest frequency of diarrhea-related ED visits but also demonstrated the highest levels of related web-based search activity. This may be attributable to 2 converging factors: the higher symptom burden in this age group and their growing reliance on internet-based health resources. Whereas younger adults are more inclined to seek health information through social media platforms [42], older adults tend to prefer structured, one-way sources such as search engines, further reinforcing their visibility in search volume data.

The overlap of our study period with the early phase of the COVID-19 pandemic (2020-2021) may have influenced both internet search behavior and ED usage patterns. During this time, public awareness of gastrointestinal symptoms, some of which overlap with COVID-19 manifestations, may be heightened due to widespread media coverage and health messaging. This may have led to increased search volume for symptom-related terms such as “diarrhea” or “upset stomach,” even in the absence of actual illness, thereby introducing potential noise into search trends [44]. Furthermore, studies have shown that many individuals delayed or avoided ED visits for non–COVID-19 conditions due to fear of infection or changes in health care access during the pandemic [45,46]. These behavioral shifts may have altered the typical relationship between symptom occurrence, web-based search activity, and clinical visits. As such, caution is warranted when interpreting our results from the pandemic period, as search and health care usage behaviors were likely atypical during this time.

### Limitations

Our study has several limitations. First, RSVs may not fully capture the motivations behind individual searches, as users may be influenced by seasonal trends, media coverage, or general health concerns rather than actual symptoms. While social media platforms like YouTube are increasingly used by younger populations for health-related information, our study could not incorporate such data due to limitations in available sources. Furthermore, potential confounding factors such as pandemics or public health campaigns were not controlled for. Nevertheless, previous research supports that individuals often turn to search engines early in the symptom experience, suggesting that, despite these limitations, RSVs remain a valuable proxy for monitoring population-level health trends when interpreted cautiously. Along with that, NAVER is the dominant search engine in South Korea; our findings may not be directly generalizable to other regions where different platforms are more commonly used. However, the underlying methodology—leveraging AI-generated symptom synonyms and analyzing their temporal relationship with health outcomes—can be adapted to platforms. Future cross-platform comparative studies are warranted to examine the consistency and reliability of search behavior across different sociotechnical contexts. In addition, the study does not account for potential misspellings or alternative phrasings, which could have resulted in underestimating actual search volumes. These factors may have affected the comprehensiveness of the data and the overall accuracy of the findings. Second, using ChatGPT carries the risk of providing incorrect information, exhibiting algorithmic bias, and perpetuating biases present in its training data [21,47]. Nevertheless, GPT-4 demonstrated higher response validity than other AI chatbots, with a correct answer rate of 82.2% in medical knowledge, indicating its potential utility in medical education [48,49]. This study used search terms suggested by ChatGPT, rather than relying solely on scientific MeSH (Medical Subject Headings) terms, to include a wide range of potential search terms. ChatGPT uses a large language model and the deep learning architecture developed by OpenAI to search across an immense collection of real-time data and is designed to respond to natural language queries with almost identical words used by general people [22]. To address this issue, we assembled a multidisciplinary team comprising AI data scientists, epidemiologists, and a medical doctor as coauthors for our study, fostering thorough discussions to guarantee a balanced and thorough analysis of the data. Third, the NEDIS database includes data from only 40% of EDs (211 out of 522), primarily large urban hospitals [50]. This coverage may introduce a bias toward urban populations, underrepresenting rural and smaller health care facilities. Patient populations in rural areas tend to be older, with higher rates of chronic conditions, which could result in different health-seeking behaviors and diagnostic patterns compared to urban populations [51]. In addition, rural EDs often rely on general emergency physicians who may operate with few diagnostic resources and less specialized equipment. These disparities could influence the accuracy and completeness of data reported to NEDIS. Consequently, the findings of this study may not fully reflect health care usage trends in rural areas or smaller health care facilities. Fourth, the 4-year gap between the data collection period (2017–2021) and manuscript submission (2024) reflects the rapidly evolving nature of digital health-seeking behaviors. The emergence and widespread use of large language models, such as ChatGPT, may have altered how individuals search for health information on the web. As conversational AI tools become more prevalent, future studies should investigate how these shifts affect search engine usage patterns. While our findings remain relevant for understanding pre-AI behaviors, longitudinal comparisons with post-AI data could enhance digital surveillance approaches. Fifth, the COVID-19 pandemic has profoundly influenced health care usage and public health awareness. Behaviors such as an increased reliance on telemedicine, a heightened focus on infectious disease symptoms, and a greater public familiarity with digital health solutions have altered the generalizability of findings based on pre- and early-pandemic data. However, as the pandemic subsided, public interest in digital health solutions and symptom searching did not sustain at peak levels, potentially impacting the applicability of pre-2024 search patterns to current contexts [44,52].

Despite these limitations, our study provides empirical evidence supporting the utility of ChatGPT-generated search terms in identifying colloquial synonyms that are not captured by MeSH terms. By demonstrating strong correlations between the RSVs of these terms, we highlight ChatGPT’s potential to complement traditional search term generation methodologies in health informatics. This approach addresses the limitations of traditional methods, including Google Trends studies, which often rely on static and predefined keywords. By contrast, our approach uses ChatGPT to dynamically adapt to changes in search behaviors and emerging terms. This not only reduces the reliance on manual efforts but also ensures that the selected terms are reflective of diverse and evolving search behaviors. In addition, we incorporated a validation process using RSV data to ensure the generated terms were both relevant and representative of real-world users’ search behaviors. By demonstrating significant correlations and timely associations between specific diarrhea-related search terms and ED visits, the study validates the potential of AI to enhance symptom surveillance. Moreover, our analyses of the age-based distribution of search engine users and ED visitors provide valuable demographic insights, highlighting which age groups are most likely to seek health information on the web and require medical care. Unlike global platforms such as Google, NAVER reflects local cultural and linguistic nuances, making it particularly well-suited for capturing region-specific search behaviors [53]. In addition, our study offers insights into how internet-based syndromic surveillance may help address both the digital divide and rural underrepresentation in public health data. Rural populations often face limited access to broadband, digital tools, and clinical infrastructure, which can hinder both their web-based health engagement and inclusion in clinical surveillance systems [54]. However, symptom search data from NAVER can serve as a supplementary data stream, particularly in areas where ED data are incomplete or unavailable [55]. In addition, by using ChatGPT to generate colloquial and culturally relevant synonyms, our study enhances the inclusivity of web-based surveillance. Previous research has shown that laypeople, especially those in older or rural populations, often use nonstandard language to describe symptoms [56]. ChatGPT’s ability to capture such expressions increases the sensitivity of surveillance models across linguistic and educational divides.

### Conclusions

The study demonstrates that the RSVs for diarrhea synonyms such as “watery diarrhea,” “upset stomach,” and “acute enteritis” are significantly associated with ED visits for diarrhea-related symptoms. These findings suggest that web search trends can effectively serve as an early indicator for increased ED visits, offering a valuable tool for real-time syndromic surveillance. For clinicians, this methodology can be integrated into daily practice to anticipate surges in diarrhea-related cases. By monitoring search volumes for these key terms, health care providers can better allocate resources, prepare for potential outbreaks, and inform patient care strategies before an uptick in physical visits occurs. Furthermore, this approach is adaptable across different languages and countries by leveraging the leading search engines in respective regions, making it a promising tool for global health monitoring.

## Appendix

Multimedia Appendix 1 Additional information. Prompt for data acquisition of synonyms for diarrhea; the 4 Korean search terms, including 1 symptom and 3 synonyms; correlation between diarrhea-related illness (NEDIS) and diarrhea related searches (NAVER) from January 2017 to December 2021; and fitted parameters of ARIMAX models for the associations between relative search volumes of symptoms and emergency department visits across lag periods (0 to 2 weeks).

## Abbreviations

AI: artificial intelligence
AICc: corrected Akaike Information Criterion
ARIMAX: Autoregressive Integrated Moving Average with Exogenous Variables
ED: emergency department
MeSH: Medical Subject Headings
NEDIS: National Emergency Department Information System
RSV: relative search volume
UMLS: Unified Medical Language System

## Data Availability

The datasets generated or analyzed during this research process are not publicly available due to South Korean privacy laws. However, you can apply for access to the data via the National Emergency Department Information System (NEDIS) or the NEVER Data Lab.

## References

[1] Park J, Yeo Y, Ji Y, Kim B, Han K, Cha W, Son M, Jeon H, Park J, Shin D (2022). Factors associated with emergency department visits and consequent hospitalization and death in Korea using a population-based national health database. Healthcare (Basel).

[2] Weiss A, Jiang H (2021). Most frequent reasons for emergency department visits, 2018. HCUP Statistical Brief.

[3] Bellido-Blasco J, Arnedo-Pena A (2011). Epidemiology of infectious diarrhea. Environ Health.

[4] Perin J, Mulick A, Yeung D, Villavicencio F, Lopez G, Strong KL, Prieto-Merino D, Cousens S, Black RE, Liu L (2022). Global, regional, and national causes of under-5 mortality in 2000-19: an updated systematic analysis with implications for the sustainable development goals. Lancet Child Adolesc Health.

[5] Aoun L, Lakkis N, Antoun J (2020). Prevalence and outcomes of web-based health information seeking for acute symptoms: cross-sectional study. J Med Internet Res.

[6] Michie S, Yardley L, West R, Patrick K, Greaves F (2017). Developing and evaluating digital interventions to promote behavior change in health and health care: recommendations resulting from an international workshop. J Med Internet Res.

[7] Hswen Y, Zhang A, Ventelou B (2021). Estimation of asthma symptom onset using internet search queries: lag-time series analysis. JMIR Public Health Surveill.

[8] Halford EA, Lake AM, Gould MS (2020). Google searches for suicide and suicide risk factors in the early stages of the COVID-19 pandemic. PLoS One.

[9] Bahk GJ, Kim YS, Park MS (2015). Use of internet search queries to enhance surveillance of foodborne illness. Emerg Infect Dis.

[10] Yang S, Santillana M, Kou SC (2015). Accurate estimation of influenza epidemics using google search data via ARGO. Proc Natl Acad Sci U S A.

[11] Ginsberg J, Mohebbi MH, Patel RS, Brammer L, Smolinski MS, Brilliant L (2009). Detecting influenza epidemics using search engine query data. Nature.

[12] Yang L, Zhang T, Han X, Yang J, Sun Y, Ma L, Chen J, Li Y, Lai S, Li W, Feng L, Yang W (2023). Influenza epidemic trend surveillance and prediction based on search engine data: deep learning model study. J Med Internet Res.

[13] Ondrikova N, Harris JP, Douglas A, Hughes HE, Iturriza-Gomara M, Vivancos R, Elliot AJ, Cunliffe NA, Clough HE (2023). Predicting norovirus in England using existing and emerging syndromic data: infodemiology study. J Med Internet Res.

[14] Olawade DB, Wada OJ, David-Olawade AC, Kunonga E, Abaire O, Ling J (2023). Using artificial intelligence to improve public health: a narrative review. Front Public Health.

[15] Hyllestad S, Amato E, Nygård K, Vold L, Aavitsland P (2021). The effectiveness of syndromic surveillance for the early detection of waterborne outbreaks: a systematic review. BMC Infect Dis.

[16] Adedire O, Love NK, Hughes HE, Buchan I, Vivancos R, Elliot AJ (2024). Early detection and monitoring of gastrointestinal infections using syndromic surveillance: a systematic review. Int J Environ Res Public Health.

[17] Hassid BG, Day LW, Awad MA, Sewell JL, Osterberg EC, Breyer BN (2017). Using search engine query data to explore the epidemiology of common gastrointestinal symptoms. Dig Dis Sci.

[18] Nuti SV, Wayda B, Ranasinghe I, Wang S, Dreyer RP, Chen SI, Murugiah K (2014). The use of google trends in health care research: a systematic review. PLoS One.

[19] Salaris S, Ocagli H, Casamento A, Lanera C, Gregori D (2025). Foodborne event detection based on social media mining: a systematic review. Foods.

[20] Jeblick K, Schachtner B, Dexl J, Mittermeier A, Stüber AT, Topalis J, Weber T, Wesp P, Sabel BO, Ricke J, Ingrisch M (2024). ChatGPT makes medicine easy to swallow: an exploratory case study on simplified radiology reports. Eur Radiol.

[21] Cascella M, Montomoli J, Bellini V, Bignami E (2023). Evaluating the feasibility of ChatGPT in healthcare: an analysis of multiple clinical and research scenarios. J Med Syst.

[22] Dwivedi YK, Kshetri N, Hughes L, Slade EL, Jeyaraj A, Kar AK, Baabdullah AM, Koohang A, Raghavan V, Ahuja M, Albanna H, Albashrawi MA, Al-Busaidi AS, Balakrishnan J, Barlette Y, et al (2023). Opinion Paper: “So what if ChatGPT wrote it?” multidisciplinary perspectives on opportunities, challenges and implications of generative conversational AI for research, practice and policy. Int J Inf Manag.

[23] Lee K, Lim D, Paik J, Choi YY, Jeon J, Sung HK (2022). Suicide attempt-related emergency department visits among adolescents: a nationwide population-based study in Korea, 2016-2019. BMC Psychiatry.

[24] Ko Y, Kim HJ, Cha ES, Kim J, Lee WJ (2012). Emergency department visits due to pesticide poisoning in South Korea, 2006-2009. Clin Toxicol (Phila).

[25] Choi DH, Jung JY, Suh D, Choi JY, Lee SU, Choi YJ, Kwak YH, Kim DK (2021). Impact of the COVID-19 outbreak on trends in emergency department utilization in children: a multicenter retrospective observational study in seoul metropolitan area, Korea. J Korean Med Sci.

[26] Yang HJ, Kim GW, Kim H, Cho JS, Rho TH, Yoon HD, Lee MJ, NEDIS-CA Consortium (2015). Epidemiology and outcomes in out-of-hospital cardiac arrest: a report from the NEDIS-based cardiac arrest registry in Korea. J Korean Med Sci.

[27] Kwon C (2023). A study on major uninsured Korean medicine treatments search trends and their meanings in an online portal: using naver data lab. J Korean Med.

[28] Kim J, Han J, Chun BC (2022). Trends of internet search volumes for major depressive disorder symptoms during the COVID-19 pandemic in Korea: an interrupted time-series analysis. J Korean Med Sci.

[29] Jimenez A, Santed-Germán MA, Ramos V (2020). Google searches and suicide rates in Spain, 2004-2013: correlation study. JMIR Public Health Surveill.

[30] Gore JI, Surawicz C (2003). Severe acute diarrhea. Gastroenterol Clin North Am.

[31] Nelson BK (1998). Statistical methodology: V. Time series analysis using autoregressive integrated moving average (ARIMA) models. Acad Emerg Med.

[32] Hyndman RJ, Khandakar Y (2008). Automatic time series forecasting: the forecast package for R. J Stat Softw.

[33] Kwiatkowski D, Phillips PC, Schmidt P, Shin Y (1992). Testing the null hypothesis of stationarity against the alternative of a unit root. J Econom.

[34] Ekström A, Kurland L, Farrokhnia N, Castrén M, Nordberg M (2015). Forecasting emergency department visits using internet data. Ann Emerg Med.

[35] Azqul M, Krakower D, Kalim S, Merchant RC (2024). Emergency department visits in the United States by adults with a complaint of diarrhea (2016-2021). J Am Coll Emerg Physicians Open.

[36] DuPont HL (1997). Guidelines on acute infectious diarrhea in adults. The practice parameters committee of the American college of gastroenterology. Am J Gastroenterol.

[37] Park SI, Giannella RA (1993). Approach to the adult patient with acute diarrhea. Gastroenterol Clin North Am.

[38] Bolia R (2017). Approach to "Upset Stomach". Indian J Pediatr.

[39] Eysenbach G, Köhler C (2002). How do consumers search for and appraise health information on the world wide web? Qualitative study using focus groups, usability tests, and in-depth interviews. BMJ.

[40] Bachl M, Link E, Mangold F, Stier S (2024). Search engine use for health-related purposes: behavioral data on online health information-seeking in Germany. Health Commun.

[41] Moon Y (2021). Survey on the internet usage in 2021, national information society agency, Daegu. NIA-VIII-RSE-C-21061.

[42] Lim MSC, Molenaar A, Brennan L, Reid M, McCaffrey T (2022). Young adults' use of different social media platforms for health information: insights from web-based conversations. J Med Internet Res.

[43] Hardt JH, Hollis-Sawyer L (2007). Older adults seeking healthcare information on the internet. Educ Gerontol.

[44] van Kessel R, Kyriopoulos I, Wong BLH, Mossialos E (2023). The Effect of the COVID-19 Pandemic on Digital Health–Seeking Behavior: Big Data Interrupted Time-Series Analysis of Google Trendsv. J Med Internet Res.

[45] Luciani LG, Mattevi D, Cai T, Giusti G, Proietti S, Malossini G (2020). Teleurology in the time of covid-19 pandemic: here to stay?. Urology.

[46] Czeisler MÉ, Marynak K, Clarke KE, Salah Z, Shakya I, Thierry JM, Ali N, McMillan H, Wiley JF, Weaver MD, Czeisler CA, Rajaratnam SM, Howard ME (2020). Delay or avoidance of medical care because of COVID-19-related concerns - United States, June 2020. MMWR Morb Mortal Wkly Rep.

[47] Shen Y, Heacock L, Elias J, Hentel KD, Reig B, Shih G, Moy L (2023). ChatGPT and other large language models are double-edged swords. Radiology.

[48] Mohammad-Rahimi H, Ourang SA, Pourhoseingholi MA, Dianat O, Dummer PMH, Nosrat A (2024). Validity and reliability of artificial intelligence chatbots as public sources of information on endodontics. Int Endod J.

[49] Torres-Zegarra BC, Rios-Garcia W, Ñaña-Cordova AM, Arteaga-Cisneros KF, Chalco XCB, Ordoñez MAB, Rios CJG, Godoy CAR, Quezada KLTP, Gutierrez-Arratia JD, Flores-Cohaila JA (2023). Performance of chatGPT, bard, claude, and bing on the peruvian national licensing medical examination: a cross-sectional study. J Educ Eval Health Prof.

[50] Kim S, Kim S, Choi BY, Park B (2024). Trends for syndromic surveillance of norovirus in emergency department data based on chief complaints. J Infect Dis.

[51] Moon BH, Lee SM, Oh M, Ryu HH, Heo T (2016). Analysis of emergency department utilization rate by region, emergency medical center, and hospital type. J Korean Soc Emerg Med.

[52] Alanezi F (2024). Factors influencing patients’ engagement with ChatGPT for accessing health-related information. Crit Public Health.

[53] Konieczny P, Danabayev K, Kennedy K, Varpahovskis E (2023). Information literacy in South Korea: similarities and differences between Korean and international students’ research trajectories. Asia Pac J Educ.

[54] Charles-Smith LE, Reynolds TL, Cameron MA, Conway M, Lau EHY, Olsen JM, Pavlin JA, Shigematsu M, Streichert LC, Suda KJ, Corley CD (2015). Using social media for actionable disease surveillance and outbreak management: a systematic literature review. PLoS One.

[55] Carneiro H, Mylonakis E (2009). Google trends: a web-based tool for real-time surveillance of disease outbreaks. Clin Infect Dis.

[56] Zhang Y, Milinovich G, Xu Z, Bambrick H, Mengersen K, Tong S, Hu W (2017). Monitoring pertussis infections using internet search queries. Sci Rep.

