# Supplementary material for: Temporal Association Between ChatGPT-Generated Diarrhea Synonyms in Internet Search Queries and Emergency Department Visits for Diarrhea-Related Symptoms in South Korea: Exploratory Study
Source: J Med Internet Res. 2025 May 22;27:e65101. doi: 10.2196/65101 (PMC12141962; doi:10.2196/65101)
Supplement: Multimedia Appendix 1 [file jmir_v27i1e65101_app1.docx]

Figure S1. Prompt for data acquisition of synonyms for diarrhea


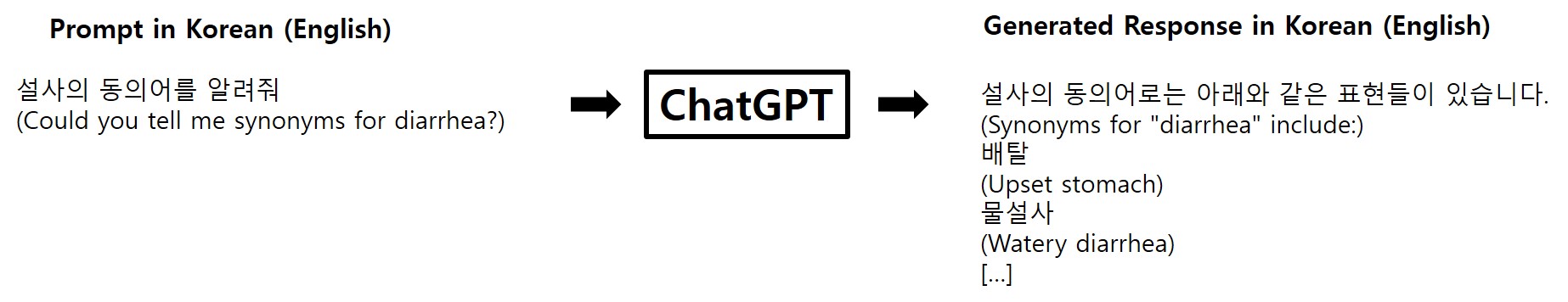


Table S1. The four Korean search terms, including 1 symptom and 3 synonyms

| Symptoms in Korea (English) | Synonyms of symptoms in Korea (English) |
| --- | --- |
| 설사(Diarrhea) | 배탈(Upset stomach), 물 설사(Watery diarrhea), 급성 장염(Acute enteritis) |

Table S2. Correlation between diarrhea-related illness (NEDIS) and diarrhea related searches (NAVER) from January 2017 to December 2021

| Diarrhea and its synonyms  from NAVER | Diarrhea-related illness obtained from the NEDIS | | | | | | | | | | | | | | | |
| --- | --- | --- | --- | --- | --- | --- | --- | --- | --- | --- | --- | --- | --- | --- | --- | --- |
|  | Diarrhea (C0011991) | | *P*-value* | | Acute diarrhea  (C0740441) | | *P*-value* | | Watery diarrhea (C0239182) | | *P*-value* | | Vomit with diarrhea (C0474496) | | *P*-value* | |
|  | r | *P*-value | 1 week | 2 weeks | r | *P*-value | 1 week | 2 weeks | r | *P*-value | 1 week | 2 weeks | r | *P*-value | 1 week | 2 weeks |
| **Diarrhea** | 0.20 | 0.001 | 0.209 | 0.606 | 0.17 | 0.005 | 0.313 | 0.707 | 0.10 | 0.112 | 0.958 | 0.517 | 0.07 | 0.275 | 0.999 | 0.741 |
| **Upset stomach** | 0.41 | <.001 | <.001 | 0.001 | 0.38 | <.001 | <.001 | 0.001 | 0.14 | 0.026 | 0.381 | 0.691 | 0.21 | <.001 | 0.042 | 0.086 |
| **Watery diarrhea** | 0.30 | <.001 | 0.141 | 0.330 | 0.23 | <.001 | 0.186 | 0.840 | 0.51 | <.001 | 0.002 | 0.009 | 0.08 | 0.187 | 0.509 | 0.617 |
| **Acute enteritis** | 0.22 | <.001 | 0.023 | 0.087 | 0.16 | <.001 | 0.043 | 0.216 | 0.32 | <.001 | 0.004 | 0.011 | -0.02 | 0.713 | 0.659 | 0.965 |

*The Granger causality test was utilized to determine the correlation for searches 1 to 2 weeks prior to emergency room visit

Table S3. Fitted parameters of ARIMAX models for the associations between RSV symptoms and ED visits across lag periods (0 to 2 weeks)

| **RSV symptoms** | **ED visits** | **Model** | **Lag** | **Coef** | **SE** | **P-value** | **MAPE** | **sMAPE** |
| --- | --- | --- | --- | --- | --- | --- | --- | --- |
| Upset stomach | Diarrhea | ARIMAX(0,1,1)(0,0,0)[52] | 0 | 22.542 | 5.033 | <.001 | 0.161 | 0.187 |
|  |  | ARIMAX(1,1,2)(0,0,0)[52] | 1 | 14.605 | 5.560 | <.001 | 0.128 | 0.134 |
|  |  | ARIMAX(0,1,3)(0,0,0)[52] | 2 | 16.215 | 4.813 | <.001 | 0.123 | 0.137 |
| Watery diarrhea | Watery diarrhea | ARIMAX(0,1,1)(0,0,0)[52] | 0 | 3.280 | 0.385 | <.001 | 0.648 | 1.018 |
|  |  | ARIMAX(1,0,1)(0,0,0)[52] | 1 | 1.848 | 0.153 | <.001 | 0.257 | 0.266 |
|  |  | ARIMAX(1,0,1)(0,0,0)[52] | 2 | 1.114 | 0.500 | 0.027 | 0.294 | 0.269 |
| Acute enteritis | Watery diarrhea | ARIMAX(1,0,1)(0,0,0)[52] | 0 | 0.385 | 0.276 | 0.163 | 0.361 | 0.304 |
|  |  | ARIMAX(3,0,2)(0,0,0)[52] | 1 | 1.123 | 0.141 | <.001 | 0.334 | 0.306 |
|  |  | ARIMAX(3,0,2)(0,0,0)[52] | 2 | 1.034 | 0.200 | <.001 | 0.321 | 0.327 |

ARIMAX, Autoregressive Integrated Moving Average with Exogenous variables; RSV, relative search volume; ED, emergency department; Coef, coefficients; SE, standard errors; MAPE, Mean absolute percentage error; sMAPE, Symmetric mean absolute percentage error
